# Residential Exposure to Traffic and Spontaneous Abortion

**DOI:** 10.1289/ehp.0900943

**Published:** 2009-08-26

**Authors:** Rochelle S. Green, Brian Malig, Gayle C. Windham, Laura Fenster, Bart Ostro, Shanna Swan

**Affiliations:** 1 Office of Environmental Health Hazard Assessment, California Environmental Protection Agency, Oakland, California, USA; 2 Division of Environmental and Occupational Disease Control, California Department of Public Health, Richmond, California, USA; 3 Department of Obstetrics and Gynecology, University of Rochester School of Medicine and Dentistry, Rochester, New York, USA

**Keywords:** air pollution, cohort, geographic information system, pregnancy, spontaneous abortion, vehicular traffic

## Abstract

**Background:**

Studies have shown associations between air pollution or traffic exposure and adverse birth outcomes, such as low birth weight. However, very few studies have examined the effect of traffic emissions on spontaneous abortion (SAB).

**Objective:**

The goal of this study was to determine whether residential exposure to vehicular traffic was associated with SAB.

**Methods:**

Pregnant women from a prepaid health plan in California were recruited into a prospective cohort study in 1990–1991. Three measures of traffic exposure were constructed for the 4,979 participants using annual average daily traffic (AADT) counts near each residence and distance from residence to major roads. SAB was examined in relation to the traffic exposure measures using logistic regression adjusting for a number of demographic and lifestyle variables.

**Results:**

Of the traffic measures, maximum annual average traffic within 50 m showed the strongest association with SAB, although it was not statistically significant. The adjusted odds ratio (AOR) for the top 90th percentile (AADT greater than 15,199) versus the bottom 75th percentile (AADT = 0–1,089) was 1.18 [95% confidence interval (CI), 0.87–1.60]. However, subgroup analyses showed statistically significant associations for traffic with SAB among African Americans (AOR = 3.11; 95% CI, 1.26–7.66) and nonsmokers (AOR = 1.47; 95% CI, 1.07–2.04).

**Conclusion:**

In this cohort, living within 50 m of a road with AADT of 15,200 or more was significantly associated with SAB among African Americans and nonsmokers. Further research is needed to confirm these results and possibly elucidate the mechanisms responsible for the findings.

Air pollution has been strongly associated with a number of adverse health outcomes, ranging from respiratory symptoms to cardiopulmonary and all-cause mortality (Garshick et al. 2003; Ostro et al. 2006; Pope et al. 2002). Several studies also examined the effects of air pollution on pregnancy, providing evidence that exposure to air pollution is associated with poor birth outcomes, such as low birth weight (Maisonet et al. 2001; Ritz and Yu 1999), small-for-gestational-age (Parker et al. 2005), and preterm birth (Ritz et al. 2000; Xu et al. 1995). These studies are relatively common because they use birth outcome data routinely collected by governmental entities. In contrast, data on spontaneous abortion (SAB) that occurs 20 weeks or earlier into gestation are not routinely collected. The literature on the relationship between air pollution and SAB is thus sparse, and the studies have yielded inconclusive results (Hansteen et al. 1996; Hemminki and Niemi 1982). Environmental tobacco smoke (ETS), an exposure that contains many of the same components as vehicle exhaust, has been associated with an increased risk of SAB (George et al. 2006; Windham et al. 1999), and it seems reasonable that exposure to traffic pollutants could have a similar relationship. The Kaiser Pregnancy Outcomes Study cohort, which represents over 5,000 pregnancies and includes data on many potential confounders, provides a unique opportunity to investigate this hypothesis.

We chose to examine the association between SAB and air pollution using residential proximity to traffic as a proxy for individual exposure to traffic pollutants. This approach has been used in a number of other studies assessing the effects of air pollution on other health outcomes (Garshick et al. 2003; Kim et al. 2004) and has been found to correlate with actual measured levels of pollutants at study sites (Gauderman et al. 2005; Zhu et al. 2002). In this study, we evaluated the relationship between SAB and traffic exposure derived from the residence of each participant.

## Methods

### Subject recruitment and interview

Details of data collection have been published elsewhere (Swan et al. 1998) and are briefly summarized here. Between 1990 and 1991, pregnant women were recruited from three regional sites of Kaiser Permanente health maintenance organization in California when they called to schedule their first prenatal appointment. The Kaiser facilities were located in the following areas: the East Bay, an area east of San Francisco including Berkeley and Oakland; Santa Clara County, south of San Francisco; and San Bernardino County, east of Los Angeles. This study complied with all applicable requirements of the United States including obtaining institutional review board approval at the participating Kaiser facilities. To be eligible to participate, the pregnant women had to be at least 18 years of age, ≤ 12 weeks into their pregnancy (mean = 8 weeks) and either English or Spanish speaking.

Of the 7,881 women initially invited for participation, 5,342 women completed a computer-assisted telephone interview sometime during the first 13 weeks of pregnancy. Participants gave informed consent over the telephone. They provided information on residential, medical, and pregnancy history; demographics; employment status and occupational exposures; diet; lifestyle; and other factors. A total of 1,412 refused participation, 558 were determined to be ineligible, 268 were not pregnant upon telephone contact, and 301 women either could not be reached or did not complete their interview.

### Pregnancy outcomes

Pregnancy outcome information was obtained for 99% of those completing interviews. Outcomes were assessed from computerized hospital admission files, and, for pregnancy losses, abstracted medical records. Gestational age was assessed using each woman’s report of her last menstrual period (LMP) during interview. If that calculation was < 4 or > 45 weeks to outcome, gestational age was reassessed based on additional information. Elective abortions (*n* = 128), ectopic pregnancies (*n* = 13), and molar pregnancies (*n* = 4) were excluded from the analysis. Among the remaining nonlive births, SAB was defined as a pregnancy ending at ≤ 20 completed gestational weeks; pregnancies ending after that period were considered stillbirths. Multiple live births (*n* = 55) were treated as a single pregnancy. There were 5,144 participants available for analysis (499 SABs, 4,613 live births, and 32 stillbirths).

### Assessment of exposure. Spatial variation

Procedures for geocoding of residences were reported previously (Green et al. 2004; Waller et al. 2001). We had a residential history for each woman. We selected the residence at the time of the LMP if it differed from residence at the time of interview, because we wanted to ensure that exposure would be assessed very early in pregnancy, including the time of implantation. Only 6% of the women moved between LMP and interview. Geocoding of the residential address at LMP was performed using ArcView geographic information system (GIS) software (version 3.0a; Environmental Systems Research Institute, Redlands, CA) with StreetMap extension (Build 7), and Mapquest. We were able to successfully geocode the residences of 4,979 of the 5,144 study subjects. The addresses of the remaining women were not geocoded because of post office boxes, missing data on residence, or inability of the software to locate the address on the street maps. Those women were, on average, slightly older (by < 1 year) than the women included in the study but did not differ from them with respect to race/ethnicity, smoking status, pregnancy outcome, employment status, and socioeconomic status (SES). The geocoded residences were overlaid with a street network layer of traffic data for 1992 provided by the California Department of Transportation (CalTrans) (California Department of Transportation 1993); traffic counts were very close in time to the study period. These data provide the annual average daily traffic (AADT) for all roads of the following functional classes: principal arterial interstates, principal arterial freeways and highways, minor arterials, and major and minor collectors (Green et al. 2004). There are no traffic flow data for local residential streets. The AADT represents the average number of vehicles traveling in both directions on a discrete road segment. Using ArcView 3.2a, we determined all road segments located within a 300-m radius of each woman’s residence, as well as information regarding functional class and AADT for 1992.

Exposure to residential traffic was estimated for each woman using each of the following three traffic metrics: *a*) Maximum AADT within a specified buffer was constructed by taking the largest reported AADT of the road segments within a set radius from the residence (50 m, 100 m, and 150 m). If there were only local residential streets and no CalTrans counted segments in the buffer, we assigned a value of zero to that residence to construct percentiles of traffic volume. *b*) We estimated the road segment with the highest AADT within 150 m after application of a Gaussian weight based on distance from the residence. The weighting model, developed by Pearson et al. (2000), assumes dispersion of motor vehicle exhaust pollutants mimicking a Gaussian probability distribution such that 96% has dissipated at 500 feet (152.4 m). We chose a distance of 150 m for this metric to capture roads with very high traffic volume, such as freeways, that may have been located > 50 or 100 m from the residence. In the tables and text below, the traffic volume derived from this method is referred to as the maximum weighted traffic. *c*) We estimated the distance in meters from the participant’s residence to the nearest CalTrans-labeled arterial roadway including principal arterial interstates, principal arterial freeways and highways, and minor arterials.

#### Temporal variation

The traffic counts we used to assess the spatial variability in exposure are yearly averages; they do not take into account temporal variations in traffic patterns and meteorologic conditions, both of which can affect the ambient levels of traffic-related air pollutants. To incorporate this temporal variation in our exposure model, we also adjusted the traffic exposure counts by the average levels of nitrogen dioxide during the periconceptual period (0–29 days post-LMP) and during the second month of pregnancy (30–59 days post-LMP) (Slama et al. 2007). We chose these two windows of exposure because exposure during this time period was relevant for the outcome and would be measured at the same period of pregnancy for both the SABs and the live births (Slama et al. 2008). There were insufficient surviving cases to examine exposure during the third month. We used NO_2_ because daily measurements were available during the study period from California Air Resources Board (2008), and it has been shown to be a good marker for traffic-related air pollution (Jerrett et al. 2005). For each window of exposure, we calculated the average NO_2_ levels at the air pollution monitor closest to the residence of each woman and divided it by the average NO_2_ level for that monitor during the entire length of the study from 1990 to 1991 to obtain an adjustment factor. For example, if the NO_2_ level was 30% higher during the periconceptual period than the lifetime of the study, the adjustment factor would be 1.3. We then multiplied traffic counts by these adjustment factors and recalculated the quantiles of exposure for each of the traffic metrics. As a sensitivity analysis, we also limited our analysis to study subjects who lived within 10 km of an NO_2_ monitor (*n* = 3,787).

### Statistical analysis

We modeled the effect of each of the traffic metrics on the risk of SAB using logistic regression with SAS version 9.1 statistical software (SAS Institute Inc., Cary, NC). Maternal age, race/ethnicity, SES, cigarette smoke exposure, employment status, and stressful life events were controlled for in the final logistic regression analyses because of their impact in changing the effect estimate of traffic on SAB or because of strong *a priori* hypotheses regarding their confounder status. The logistic regression analysis was performed both with and without the temporal adjustment for NO_2_ levels. Stratified analyses by air conditioner use, season of first trimester, commute time, region, race/ethnicity, maternal age, smoking status, and employment status were performed to evaluate possible effect modification using the traffic metric (maximum traffic within 50 m) with the strongest association with SAB. For instances where effects between groups appeared visibly different and the *p-*value for the likelihood ratio test of interaction was ≤ 0.10, we evaluated the degree of departure of combined effects from additivity by calculating the relative excess risk due to interaction (RERI) and its associated 95% confidence intervals (CIs) (Hosmer and Lemeshow 1992). For example, the RERI for nonsmokers was constructed as follows: [adjusted odds ratio (AOR) for nonsmoker and highest traffic] – (AOR for nonsmoker and lowest traffic) – (AOR for smoker and highest traffic) + 1, where the reference group was smoker and lowest traffic exposure.

## Results

The incidence of SAB among the women included in this analysis was 9.6%. Most of the women were non-Hispanic white, although ethnic minorities represented 34% of the sample. Most were either married or living with their partner and reported working at time of interview. Although most women were located in several cities and suburbs east and south of San Francisco and east of Los Angeles, a few were located near the coast, where background levels of air pollution are generally lower. The distribution of SABs by demographic and exposure categories is reported in Table 1. Higher rates of SAB were associated with being ≥ 35 years of age, being African American, and having been interviewed at ≤ 8 weeks of gestation. Rates of SAB were also higher in Asians and women who recently experienced two or more stressful life events. There were slight but not statistically significant increases in risk with highest exposure to some traffic metrics, most notably maximum AADT within 50 m.

Distribution of traffic exposure was also examined by potential confounders (Table 2). Women exposed to the highest levels of traffic were more likely to be younger, report smoking during their pregnancy, be of lower SES, have recently experienced two or more stressful life events, and report working at time of interview.

Table 3 shows the AORs for SAB and traffic exposure using several traffic metrics. Among the first three metrics listed, maximum AADT (daily maximum traffic) within 50 m still showed the strongest association with SAB, although not statistically significantly, in models without the temporal adjustment (AOR for 90th vs. 75th percentile = 1.18; 95% CI, 0.87–1.60). Maximum traffic within 100 m showed stronger associations when the traffic counts were scaled using NO_2_ levels during the first (AOR = 1.23; 95% CI, 0.89–1.70) and second (AOR = 1.24; 95% CI, 0.89–1.71) months of pregnancy. The odds of SAB did not differ by distance to an arterial road. Although maximum weighted traffic within 150 m was not associated with SAB in the analysis without the temporal adjustment, adjusting for NO_2_ levels during the first month of pregnancy increased the association between SAB and that traffic metric (Table 3). There was no difference in the temporally adjusted data for any of the traffic metrics when the analysis was limited to study subjects who lived within 10 km of an air pollution monitor (data not shown).

*A priori* hypotheses regarding variables as possible effect modifiers were also assessed using the traffic metric (maximum traffic within 50 m) with the strongest association with SAB in the adjusted analyses. No effect modification was found by maternal age, employment status, season of conception, reported use of air conditioning, time spent commuting, or regional site. We did not see effect modification by region, even though the background levels of carbon monoxide, NO_2_, ozone, and particulate matter < 10 μm in aerodynamic diameter varied among the regions, with the San Bernardino area recording higher levels for most pollutants. This suggests that local variations in levels of traffic-related pollutants were more important than regional differences.

However, we did observe effect modification by race/ethnicity and smoking status, and there was a suggestion of interaction for gestational age of SAB. The AOR for African Americans in the highest traffic exposure category compared to the lowest was 3.11 (95% CI, 1.26–7.66) (Table 4), whereas among whites (and other racial/ethnic groups), traffic was not significantly associated with SAB. The excess risk of SAB in relation to traffic was about doubled in African Americans compared with whites. Furthermore, the test for interaction by race/ethnicity found that this racial/ethnic difference was statistically significant (RERI = 2.2; 95% CI, 0.7–3.6). Results were similar when the analysis was adjusted for NO_2_ levels during the first and second months of gestation.

Among women who were nonsmokers, significantly increased odds of SAB were observed in the highest traffic exposure group (AOR = 1.47; 95% CI, 1.07–2.04). Results were unchanged after the temporal adjustments. However, among women who did report smoking at some point in their pregnancy, a significantly negative association was found (AOR = 0.36; 95% CI, 0.14–0.93) when examining the highest versus the lowest traffic exposure groups. These differences in the effect of traffic between smoking groups were statistically significant (RERI for nonsmokers = 0.95; 95% CI, 0.43–1.46).

The effect estimates using maximum traffic at 50 m as the exposure metric were slightly higher for pregnancy losses at ≤ 10 weeks (AOR = 1.35; 95% CI, 0.87–2.10) versus later losses (AOR = 1.08; 95% CI, 0.73–1.60).

## Discussion

To our knowledge, this is the first published study of the effect of residential traffic exposure on the risk of SAB. We found a slightly increased risk of SAB among women residing within 50 m of a road with an AADT above the 90th percentile (or ≥ 15,200 vehicles in this study) compared with women residing near roads with maximum AADT below the 75th percentile (about 1,000 vehicles). Temporal adjustment for average NO_2_ levels during the first or second month of gestation did not change the magnitude of the association for that particular traffic metric. The increase in risk associated with traffic was stronger in African-American women than in whites and in nonsmokers than in smokers.

### Comparison with previous studies

Although the present study is the first to evaluate the association between traffic exposure and SAB, two previous studies conducted in Europe examined the association between SABs before 26 weeks of gestation, which were ascertained from hospital records, and industrial air pollution. Although the Norwegian study (Hansteen et al. 1996) failed to find an association, the Finnish study (Hemminki and Niemi 1982) found an increased rate of SAB in areas with a higher mean annual level of hydrogen sulfide (> 4 μg/m^3^). A time-series study in Brazil (Pereira et al. 1998) found a strong association between stillbirth and both NO_2_ as an individual pollutant and an index that combined NO_2_, CO, and sulfur dioxide. SAB has also been associated with ETS, which contains many of the same chemicals as traffic exhaust (George et al. 2006; Windham et al. 1999).

### Biological mechanisms

There are biologically plausible mechanisms by which the association between SAB and emissions from vehicular traffic could be causal. Researchers in Tehran found an association between ambient CO and both carboxyhemoglobin (COHb) and nucleated red blood cells, a sign of fetal hypoxia, in the venous cord blood of 41 newborns (Ziaei et al. 2005). The levels of COHb may interfere with the oxygen levels in the fetus, possibly leading to fetal death, as demonstrated in an animal model (Singh and Scott 1984).

Perera et al. (1999) found that ambient air pollution was significantly associated with the amount of polycyclic aromatic hydrocarbon (PAH) bound to DNA (PAH–DNA adducts) in both maternal and infant cord white blood cells. Previous studies have found associations between ambient air pollution and birth defects (Gilboa et al. 2005; Ritz et al. 2002). In our study, the effect of traffic was greater, albeit not significant, among women with SABs at ≤ 10 weeks than among those with later losses. This would be consistent with genetic damage as a mechanism, given that early SABs are more likely to have chromosomal abnormalities or birth defects (Byrne et al. 1985).

The effect of air pollution on SAB may also be mediated by reproductive effects in the male (Slama et al. 2005). Studies have shown that exposure to air pollution is associated with decrements in several measures of semen quality (De Rosa et al. 2003; Selevan et al. 2000). The SAB rate is higher if a man has > 30% of sperm showing DNA fragmentation (Evenson and Wixon 2005). More than 90% of the women in this study were either married or living with a partner, so traffic exposure at the maternal residence may be serving as a proxy for paternal exposure to traffic.

### Effect modification

Our finding of a stronger effect of traffic exposure among African-American women than among women of other racial/ethnic groups is based on a relatively small sample size. However, it is possible that African-American women are more sensitive to the effects of air pollution than women of other ethnicities. A study of live births in the northeastern United States found that the association between CO and term low birth weight was more consistent and stronger for African-American than for white or Hispanic infants (Maisonet et al. 2001). A study of women residing in New York City found that PAHs had a significant adverse effect on birth weight and head circumference among African-American but not among Dominican women (Perera et al. 2003). Other unmeasured confounders such as nutrition may also account for increased susceptibility to air pollution among African Americans, as they had lower mean intakes of several vitamins than other groups in the Third National Health and Nutrition Examination Survey (Arab et al. 2003). Kannan et al. (2006) postulated several mechanisms by which nutrition could modify the effect of particulate matter on birth outcomes.

Regarding the effect modification by smoking, Samoli et al. (2003) found that the effect of NO_2_ on mortality was greater in cities where the smoking prevalence was lower. In a cohort study of the effect of air pollution on lung development, Gauderman et al. (2004) found that air pollutants had no effect on growth of lung function among those who had ever smoked. Furthermore, the reduction in lung function growth associated with living within 500 m of a freeway was greater in nonsmokers than smokers (Gauderman et al. 2007). Like these studies, we found an increased risk in nonsmokers, which may be attributable, in addition to the explanations offered by other researchers, to chance or unmeasured confounders. We have no explanation for the protective effect seen in smokers, perhaps because of unmeasured confounders or chance.

### Limitations

Some women may have had SABs before they could be interviewed, and they would not be included in the cohort. This would lead to left truncation of the data, which could bias the study results based on logistic regression if the exposure was related to the week of entry into the cohort (Howards et al. 2007). In our study, the average number of weeks of gestation at interview did not differ for those women with high versus low traffic exposure (8.08 weeks and 8.02 weeks, respectively). However, bias toward the null may still have occurred if women with very high traffic exposure had SABs that were so early as to not be detected. Conversely, if women with low traffic exposure were more likely to have undetected SABs, that could lead to a bias of finding an association. The GIS methods we used have some error in assigning exposure because the street line work layer used to geocode the residential addresses did not align perfectly with the street line work from CalTrans, which contains the traffic volume data. A recent analysis of traffic exposure at child care facilities in Los Angeles County suggested that errors resulting from unreconciled data produced a modest bias in terms of aggregated number of facilities at risk (Ong et al. 2006). We assumed that these misalignment errors would be nondifferential with respect to pregnancy outcome.

Although we were able to construct a variety of traffic metrics, there were some limitations to the traffic database we used in this study. For example, we were unable to construct a metric for traffic density (sum of traffic on all roads within a buffer) because the CalTrans AADT data is reported for road segments, not complete roads. Therefore, if we had summed the traffic on all segments within a specified buffer, we would have overcounted traffic on some streets that comprised multiple segments. However, in a study of residential traffic and children’s respiratory health (Kim et al. 2008), two traffic metrics, traffic density and maximum AADT within 150 m, were similarly associated with current asthma and with bronchitis.

### Strengths

In this prospective cohort study we collected detailed interview information directly from the study participants and included information about demographics, lifestyle factors, work exposures, and residential history. We were able to directly control for SES, which was related to traffic exposure. Also, we had individual-level information about air conditioning use, commute times, and mode of transportation, which allowed us to evaluate these factors as potential confounders and effect modifiers.

We had a complete residential history for each woman; therefore we were able to select the residence at the time of the LMP if it differed from residence at the time of interview. GIS methods allowed us to measure traffic exposure within a few meters of the women’s residences, which allowed us to more accurately assess exposure to traffic-related pollutants than central site monitors alone.

## Conclusions

In this cohort, living within 50 m of a road with maximum AADT of 15,200 (top 10th percentile) or more was significantly associated with SAB among African Americans and nonsmokers. These associations occurred in areas with both high and low regional air pollution. This study adds another outcome to the increasing body of literature on the adverse reproductive health effects of exposure to air pollution from vehicular traffic. Further studies of SAB in other pregnancy cohorts, with larger sample sizes than the current one, particularly of nonwhite racial groups, are needed to confirm these findings and shed light on mechanism.

## Figures and Tables

**Table 1:** Proportion of SABs and unadjusted ORs and 95% CIs, by demographic and exposure variable categories.

Variable	No.	Cases	Percent SAB	Unadjusted OR (95% CI)
Total	4,979	479	9.62	

Mother’s age (years)				
18–24	1,351	105	7.77	1
25–34	3,084	280	9.08	1.21 (0.96–1.52)
> 34	544	94	17.28	2.52 (1.88–3.38)

Race/ethnicity				
Black	314	40	12.74	1.44 (1.02–2.02)
Hispanic/Latina	925	75	8.11	0.86 (0.67–1.12)
Asian	461	56	12.15	1.31 (0.97–1.77)
Non-Hispanic white	3,275	307	9.37	1

Marital status				
Married/living together	4,566	435	9.53	1
Separated/divorced/widowed	110	16	14.55	1.58 (0.93–2.72)
Never married	301	28	9.30	0.99 (0.66–1.46)

Week of pregnancy at interview				
0–8	3,188	350	10.98	1
> 8	1,781	128	7.19	0.63 (0.51–0.77)

Working at interview				
Yes	3,740	382	10.21	1
No	1,237	96	7.76	0.74 (0.59–0.93)

Mother’s education				
Up to high school grad/GED	2,072	183	8.83	1
Some college/tech school	1,582	157	9.92	1.13 (0.91–1.41)
College grad and above	1,320	138	10.45	1.2 (0.95–1.50)

Cigarette exposure				
Nonsmoker	4,056	387	9.54	1
Smoked at LMP or interview	907	92	10.14	1.09 (0.87–1.38)

SES				
Low income/low education	724	61	8.43	0.87 (0.66–1.15)
High income/high education	644	60	9.32	1.00 (0.75–1.31)
Other	3,496	348	9.95	1

Recent stressful life events				
0–1	3,182	289	9.08	1
≥ 2	1,797	190	10.57	1.18 (0.98–1.43)

Pregnancy history				
First pregnancy	1,266	109	8.61	1
Previous pregnancy, no SAB	2,690	251	9.33	1.15 (0.91–1.45)
One previous SAB	786	78	9.92	1.19 (0.88–1.60)
Two or more previous SABs	233	41	17.60	2.49 (1.71–3.63)

Maximum daily traffic within 50 m^a^ [percentile (range)]				
0–74th (0–1,089)	3,734	355	9.51	1
75–89th (1,090–15,199)	741	65	8.77	0.92 (0.69–1.21)
≥ 90th (≥15,200)	504	59	11.71	1.26 (0.94–1.69)

Maximum daily traffic within 100 m^a^ [percentile (range)])				
0–50th (0)	2,530	238	9.41	1
51–74th (1–11,499)	1,203	110	9.14	0.97 (0.77–1.23)
75–89th (11,500–25,799)	746	77	10.32	1.11 (0.85–1.45)
≥ 90th (≥25,800)	500	54	10.80	1.17 (0.85–1.59)

Maximum daily traffic within 150 m^a^ [percentile (range)])				
0–49th (0–5,269)	2,488	241	9.69	1
50–74th (5,270–17,999)	1,233	122	9.89	1.02 (0.81–1.29)
75–89th (18,000–33,199)	757	67	8.85	0.91 (0.68–1.20)
≥ 90th (≥33,200)	501	49	9.78	1.01 (0.73–1.40)

Distance to nearest arterial (m)				
0–50	882	92	10.43	1.06 (0.80–1.41)
51–100	783	73	9.32	0.93 (0.69–1.27)
101–200	1,196	105	8.78	0.87 (0.67–1.15)
201–300	868	85	9.79	0.99 (0.74–1.32)
> 300	1,250	124	9.92	1

Maximum weighted traffic within 150 m (percentile)				
0–50th	2,489	233	9.36	1
51–74th	1,245	122	9.80	1.04 (0.83–1.31)
75th–89th	747	72	9.64	1.03 (0.78–1.36)
≥ 90th	498	53	10.64	1.15 (0.84–1.58)

GED, general education degree.

a Local roads were assigned a value of zero.

**Table 2:** Sociodemographic characteristics and exposures among women by traffic exposure categories (%).

	Percentile for maximum daily traffic within 50 m^a^ (range in AADT)		
	0–74th (0–1,089) (*n* = 3,734)	75th–89th (1,090–15,199) (*n* = 741)	≥ 90th (≥15,200) (*n* = 504)
Mother’s age (years)			
18–24	26.14	27.26	34.33
25–34	62.88	62.35	54.37
≥ 35	10.98	10.39	11.31

Race/ethnicity			
Black	6.16	6.76	6.77
Hispanic/Latina	18.03	20.68	19.72
Asian	9.62	6.89	10.16
Non-Hispanic white	66.19	65.68	63.35

Week of pregnancy at interview			
0–8	64.50	63.65	62.35
> 8	35.50	36.35	37.65

Working at interview			
Working	74.95	73.65	78.77
Not working	25.05	26.35	21.23

Mother’s education			
Up to high school grad/GED	41.14	44.38	41.47
Some college/tech school	31.44	31.66	34.72
College grad and above	27.42	23.95	23.81

Cigarette exposure			
Nonsmoker	82.77	78.73	78.37
Smoked at LMP or interview	17.23	21.27	21.63

SES			
Low income/low education	13.99	17.97	17.00
High income/high education	14.05	11.28	10.12
Other	71.96	70.75	72.87

Recent stressful life events			
0–1	64.78	64.51	56.55
≥ 2	35.22	35.49	43.45

Pregnancy history			
First pregnancy	25.24	25.37	27.09
Previous pregnancy, no SAB	54.45	52.9	52.99
One previous SAB	15.68	17.41	14.34
Two or more previous SABs	4.64	4.32	5.58

GED, general education degree.

a Local roads were assigned a value of zero.

**Table 3:** Effects of traffic exposure on SAB using different traffic metrics.

Traffic metric	No.	AOR^a^ (95% CI)	AOR^a^ after traffic scaled using NO_2_ 0–29 days after LMP (95% CI)	AOR^a^ after traffic scaled using NO_2_ 30–59 days after LMP (95% CI)
Maximum daily traffic within 50 m^b^ [percentile (range)]				
0–74th (0–1,089)	3,734	1	1	1
75–89th (1,090–15,199)	741	0.91 (0.68–1.21)	1.04 (0.78–1.38)	1.02 (0.76–1.36)
≥ 90th (≥ 15,200)	504	1.18 (0.87–1.60)	1.16 (0.84–1.59)	1.17 (0.85–1.62)

Maximum daily traffic within 100 m^b^ [percentile (range)]				
0–49th (0)	2,530	1	1	1
50–74th (1–11,499)	1,203	0.97 (0.76–1.24)	1.00 (0.78–1.28)	0.98 (0.76–1.27)
75–89th (11,500–25,799)	746	1.10 (0.84–1.46)	1.06 (0.79–1.42)	0.97 (0.72–1.32)
≥ 90th (≥ 25,800)	500	1.11 (0.80–1.54)	1.23 (0.89–1.70)	1.24 (0.89–1.71)

Maximum daily traffic within 150 m^b^ [percentile (range)]				
0–49th (0–5,269)	2,488	1	1	1
50–74th (5,270–17,999)	1,233	1.02 (0.80–1.29)	0.91 (0.71–1.17)	0.97 (0.76–1.25)
75–89th (18,000–33,199)	757	0.88 (0.66–1.18)	1.08 (0.81–1.43)	0.96 (0.71–1.29)
≥ 90th (≥ 33,200)	501	0.99 (0.71–1.38)	1.05 (0.75–1.48)	1.05 (0.74–1.47)

Distance to nearest arterial (m)^c^				
0–50	882	1.00 (0.74–1.34)	NA	NA
51–100	783	0.93 (0.68–1.26)		
101–200	1,196	0.89 (0.67–1.18)		
201–300	868	1.00 (0.75–1.35)		
> 300	1,250	1		

Maximum weighted traffic within 150 m (percentile)				
0–49th	2,489	1	1	1
50–74th	1,245	1.07 (0.85–1.36)	1.04 (0.82–1.34)	1.06 (0.83–1.36)
75th–89th	747	1.05 (0.79–1.40)	1.02 (0.76–1.38)	0.96 (0.71–1.30)
≥ 90th	498	1.08 (0.78–1.50)	1.21 (0.87–1.67)	1.13 (0.81–1.58)

NA, not applicable.

a Adjusted for maternal age, race, employment status, SES, stressful life events (0–1 vs. ≥ 2 in the preceding 6 months), and maternal smoking (at LMP or since).

b Local roads were assigned a value of zero.

c Arterial road was defined by functional class rather than by daily traffic; therefore, NO_2_ adjustment could not be computed for this traffic metric.

**Table 4:** Effects of traffic exposure^a^ on SAB (using maximum annual daily traffic within 50 m), stratified by race and smoking exposure.

Stratum	Percentile of traffic exposure	No.	AOR^b^ (95% CI)	AOR^b^ after traffic scaled using NO_2_ 0–29 days after LMP (95% CI)	AOR^b^ after traffic scaled using NO_2_ 30–59 days after LMP (95% CI)
Race/ethnicity					
African American		314			
	0–74th		1	1	1
	75–89th		0.92 (0.31–2.74)	1.06 (0.38–2.95)	0.82 (0.26–2.57)
	≥ 90th		3.11 (1.26–7.66)	3.74 (1.53–9.12)	3.32 (1.30–8.49)
Hispanic		925			
	0–74th		1	1	1
	75–89th		1.02 (0.53–1.98)	1.11 (0.59–2.12)	1.22 (0.65–2.29)
	≥ 90th		0.87 (0.40–1.91)	0.80 (0.33–1.93)	0.50 (0.18–1.44)
Asian		461			
	0–74th		1	1	1
	75–89th		0.98 (0.39–2.47)	0.90 (0.33–2.42)	0.97 (0.36–2.64)
	≥ 90th		0.73 (0.27–1.94)	0.91 (0.34–2.48)	0.91 (0.34–2.48)
Non-Hispanic white		3,275			
	0–74th		1	1	1
	75–89th		0.86 (0.61–1.25)	1.08 (0.67–1.52)	1.00 (0.70–1.44)
	≥ 90th		1.16 (0.79–1.71)	1.04 (0.69–1.57)	1.19 (0.80–1.77)

Smoking status					
Nonsmokers at time of LMP and interview		4,056			
	0–74th		1	1	1
	75–89th		1.09 (0.80–1.49)	1.26 (0.93–1.72)	1.24 (0.91–1.70)
	≥ 90th		1.47 (1.07–2.04)	1.49 (1.06–2.10)	1.51 (1.07–2.13)
Smokers, either at LMP or interview		907			
	0–74th		1	1	1
	75–89th		0.41 (0.19–0.86)	0.43 (0.20–0.93)	0.44 (0.21–0.94)
	≥ 90th		0.36 (0.14–0.93)	0.35 (0.14–0.89)	0.36 (0.14–0.92)

a Percentiles of traffic exposure based on entire study population and use same cut points as those listed in Table 3 for maximum traffic within 50 m.

b Adjusted for SES, stressful life events (0–1 vs. ≥ 2 in the preceding 6 months), employment status, maternal age, maternal smoking (at LMP or since), and/or race/ethnicity (as appropriate).
